# A Fluorescent Detection for Paraquat Based on β-CDs-Enhanced Fluorescent Gold Nanoclusters

**DOI:** 10.3390/foods10061178

**Published:** 2021-05-24

**Authors:** Hong-Xin Ren, Min-Xin Mao, Min Li, Cun-Zheng Zhang, Chi-Fang Peng, Jiang-Guo Xu, Xin-Lin Wei

**Affiliations:** 1 State Key Lab of Food Science and Technology, Jiangnan University, Wuxi 214122, China; 6180150008@stu.jiangnan.edu.cn (H.-X.R.); 6190112080@stu.jaingnan.edu.cn (M.-X.M.); 2 School of Food Science and Technology, Jiangnan University, Wuxi 214122, China; 7190112015@stu.jiangnan.edu.cn; 3 Jiangsu Key Laboratory for Food Quality and Safety-State Key Laboratory Cultivation Base, Ministry of Science and Technology, Nanjing 210014, China; zcz@jaas.ac.cn; 4 School of Food Science and Biological Engineering, Hefei University of Technology, Hefei 230009, China; 5 School of Agriculture and Biology, Shanghai Jiaotong University, Shanghai 200240, China; wxl@shnu.edu.cn

**Keywords:** gold nanoclusters, β-cyclodextrin, fluorescence enhancement, paraquat, photoinduced energy transfer

## Abstract

In this report, a fluorescent sensing method for paraquat based on gold nanoclusters (AuNCs) is proposed. It was found that paraquat could quench both glutathione-capped AuNCs (GSH-AuNCs) and β-cyclodextrin-modified GSH-AuNCs (GSH/β-CDs-AuNCs). The modification of β-CDs on the surface of GSH-AuNCs obviously enhanced the fluorescence intensity of GSH-AuNCs and improved the sensitivity of paraquat sensing more than 4-fold. This sensibilization was ascribed to the obvious fluorescence intensity enhancement of GSH-AuNCs by β-CDs and the “host–guest” interaction between paraquat and β-CDs. The fluorescence quenching was mainly due to the photoinduced energy transfer (PET) between GSH/β-CDs-AuNCs and paraquat. With the optimized β-CDs modification of the GSH-AuNC surfaces and under buffer conditions, the fluorescent detection for paraquat demonstrated a linear response in the range of 5.0–350 ng/mL with a detection limit of 1.2 ng/mL. The fluorescent method also showed high selectivity toward common pesticides. The interference from metal ions could be easily masked by ethylene diamine tetraacetic acid (EDTA). This method was applied to the measurement of paraquat-spiked water samples and good recoveries (93.6–103.8%) were obtained. The above results indicate that host molecule modification of fluorescent metal NC surfaces has high potential in the development of robust fluorescent sensors.

## 1. Introduction

Noble metal nanoclusters (NCs) with a diameter less than 2 nm fill the gap between atom and nanoparticles. As their size approaches the Fermi wavelength of metals [1], the quantum size effect produces strongly size-dependent physicochemical properties [2], including intense luminescence, discrete electronic states, redox behavior, chirality, intrinsic magnetism, etc. [1]. Among various NCs, such as AuNCs [3], AgNCs [4], CuNCs [5], Au/AgNCs [6], Au/CuNCs [7,8], and Cu/AgNCs [9], AuNCs generally display stable properties, which has facilitated their wide application in various fields [10,11,12]. In terms of surface ligands, AuNCs are generally classified into thiolated AuNCs, dendrimer- or polymer-protected AuNCs, protein-templated or protein-protected AuNCs, DNA-templated AuNCs, and small-molecule-protected AuNCs [13]. Among various AuNCs, glutathione (GSH)-stabilized AuNCs (GSH-AuNCs) have been demonstrated to have many interesting properties and have been used in the detection of heavy metal ions such as Cr^3+^ [14], Cu^2+^ [15], Hg^2+^ [16,17,18], and Ag^+^ [19,20]; thiol [21,22]; and alkaline peptide and alkaline phosphatase [23,24]. GSH-AuNCs have also been applied to detect the electron deficiency of dopamine [25]. 

Paraquat, as a wide-spectrum but toxic herbicide, is now restricted for use in various agricultural production processes. It can cause the production of high-energy oxygen free radicals and glutathione peroxidase 4 (GPX4) inactivation, and ultimately multiorgan failure and pulmonary fibrosis [26]. Some works have also reported a relationship between paraquat and increased risk for Parkinson’s disease [27]. Nevertheless, application of paraquat in limited contexts is still permitted in many developed and undeveloped countries, including such as America and Australia [28], Colombia, Uruguay [29], Bolivia [30], and Iran [31]. Although paraquat has been prohibited in China, the illegal use of paraquat still occurs. Many analytical methods for paraquat detection, including HPLC [32] and UPLC-HRMS [33], have been developed and demonstrated to be reliable, but the requirements of expensive instruments and sophisticated skills limit their application. Nanomaterial-based sensors such as enzyme inhibition [34], nanozyme-based sensors [35], aptasensors [36], immunosensors [37], and host–guest recognition [38] methods for pesticide detection have attracted great attention from researchers. However, the above methods may suffer from shortcomings such as biomacromolecule denaturation, lengthy steps, and time-consuming protocols. To date, metal-nanocluster-based enzyme-free fluorescent sensors for pesticides have been rarely reported [19,39]. For example, Lu et al. [18] found that GSH-AuNCs demonstrated aggregation-induced emission enhancement (AIEE) in the presence of silver ions and that thiram could inhibit the above AIEE effect through coordination with the silver ions. Based on this strategy, they developed a sensitive fluorescent detection method for thiram in an electronic-eye platform. Yang et al. [39] fabricated a fluorescence resonance energy transfer (FRET) system between nitrogen-doped carbon quantum dots (N-CQDs) and gold nanoclusters (AuNCs), and they further developed a ratiometric fluorescence assay for carbendazim based on the fact that carbendazim induces the aggregation of AuNCs.

**Scheme 1:**
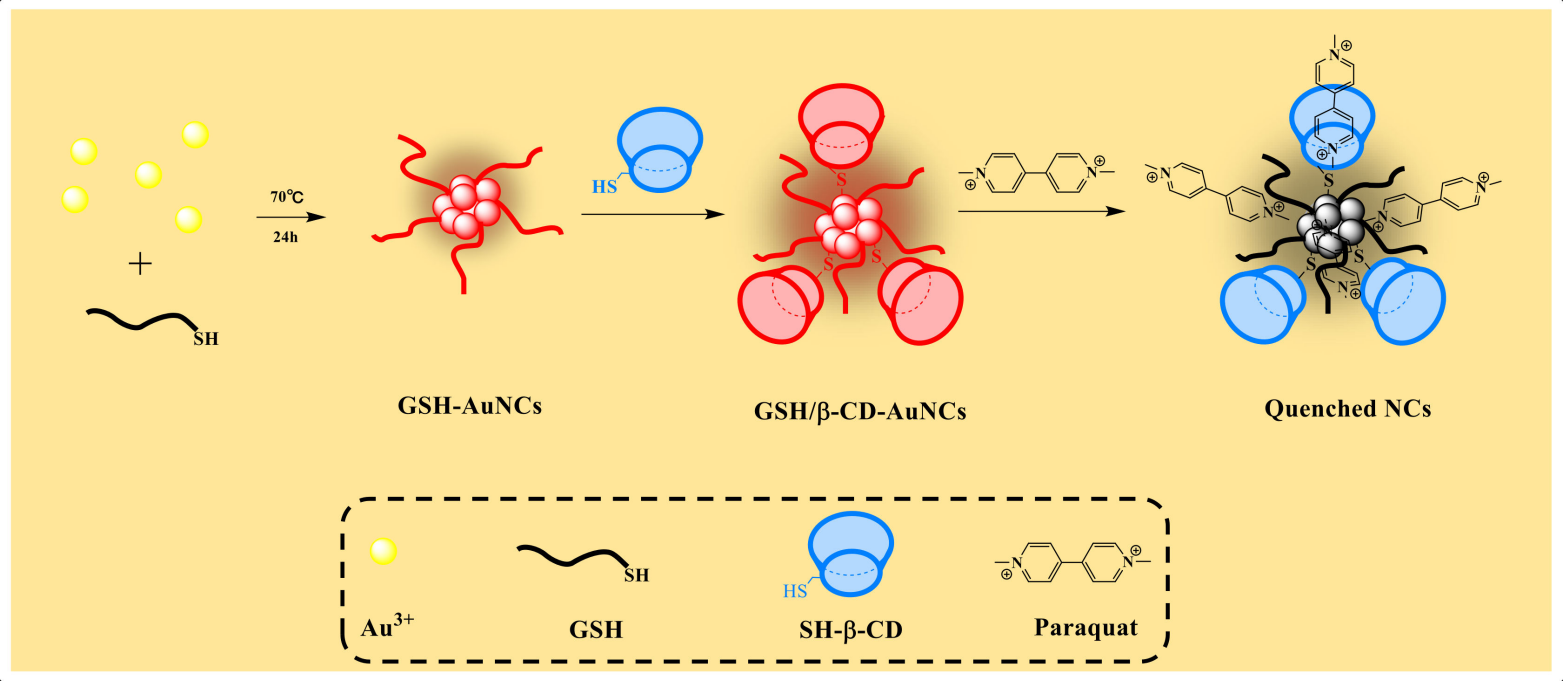
The GSH/β-CDs-AuNC synthesis method and mechanism of GSH/β-CDs-AuNC fluorescence quenching induced by paraquat.

Herein, a simple, highly sensitive, and selective fluorescent detection for paraquat was developed using GSH/β-CDs-AuNCs. To the best of our knowledge, this is the first report of gold-nanocluster-based, enzyme-free fluorescent detection for paraquat. The mechanism of paraquat-induced fluorescence quenching of GSH/β-CDs-AuNCs is shown in Scheme 1. 

## 2. Materials and Methods

### 2.1. Chemicals and Reagents

Chloroauric acid (HAuCl_4_·4H_2_O) (purity, 99%) and glutathione (GSH) (purity, 99%) were purchased from Sinopharm Chemical Reagent Co., Ltd. (Shanghai, China). Mercapto-β-cyclodextrin (98% purity) was purchased from Shandong Binzhou Zhiyuan Biotechnology Co., Ltd. All the pesticides (analytical grade) were obtained from Shanghai Pesticide Research Institute (Shanghai, China). The other utilized chemicals were of analytical grade and were used without further purification. All the solutions were prepared with ultrapure water.

### 2.2. Apparatus

The UV–vis absorbance spectrum and fluorescence spectra of GSH/β-CDs-AuNCs were obtained with a UV–1800 (Shimadzu corporation, Kyoto, Japan) and Fluorescence photometer Pro 97 (Lengguang Tech, Shanghai, China), respectively. The fluorescence lifetime of AuNCs was measured using an FLS920 model spectrofluorometer (Edinburgh Instruments Ltd., Livingstone, UK) equipped with an EPL375 pulsed laser diode. Hydrodynamic diameter and zeta potential were determined using a dynamic light scattering instrument (Zetasizer nano ZS, Malvern Corporation, Malvern, UK). Transmission electron microscopy (TEM) images were obtained using a JEM-2100 electron microscope (200 kV, Electronics Corporation, Tokyo, Japan). Fourier transform infrared spectroscopy (FTIR) spectra (at 400–4000 cm^−1^, KBr powder-pressed pellets) were collected using an iS10 spectrometer (Nicolet, Waltham, MA, USA).

### 2.3. Synthesis of SH-β-CDs and GSH-Capped AuNCs

All glassware were immersed in Aqua Regia solution (HCl/HNO_3_ = 3:1, *v*/*v*) overnight, and then washed thoroughly with ultrapure water prior to use. The GSH/β-CDs-AuNCs were synthesized via chemical reduction of HAuCl_4_ with GSH according to previous reported methods with minor modification [40,41]. Briefly, freshly prepared HAuCl_4_ aqueous solution (10 mM, 2 mL) was added to 8 mL of GSH solution (3.75 mM) at 25 °C under vigorous stirring. The color of the solution immediately changed from pale yellow to dark brown. After reacting for another 24 h at 70 °C, the solution was cooled down to room temperature. Subsequently, 10 mL of ethanol was added into the above mixture. After centrifugation at 9600× *g* for 10 min, the obtained precipitate was redissolved in 10 mL of ultrapure water. In order to obtain GSH/β-CDs-AuNCs, 10 µM to 400 µM measures of SH-β-CDs were added into the GSH-AuNCs, and the mixtures were then incubated at room temperature for 3 h. The solutions were then filtered with 3 kDa cut-off ultrafiltration tubes to remove excess ligands. The obtained GSH/β-CDs-AuNCs were then stored at 4 °C for subsequent use. The quantum yield (QY) of GSH/β-CDs-AuNCs was calculated to be 20.0%, using rhodamine 6G (QY of 95%, in ethanol) as a reference [42].

### 2.4. Fluorescent Detection of Paraquat

Sample solutions (50 µL) containing different concentrations of paraquat were added to mixture solutions which contained 50 µL of GSH/β-CDs-AuNCs (100 µM), 800 µL of Gly–NaOH buffer solution (5 mM, pH = 9.0), and 100 µL EDTA (1 mM). The fluorescence intensity was measured at 610 nm with excitation at 392 nm and was then quantified to fit the calibration curve of paraquat.

### 2.5. Validation of the Fluorescent Detection

Lake water samples were obtained from Taihu Lake (Wuxi, China). The obtained samples were spiked with a series of standard paraquat solutions and filtered through a microporous membrane prior to detection. Chinese cabbage samples were purchased from a local market. The samples were spiked with paraquat solution and homogenized. The obtained suspension was centrifuged and then filtered through a microporous membrane prior to detection.

## 3. Results and Discussion

### 3.1. Properties of the GSH/β-CDs-AuNCs

**Figure 1:**
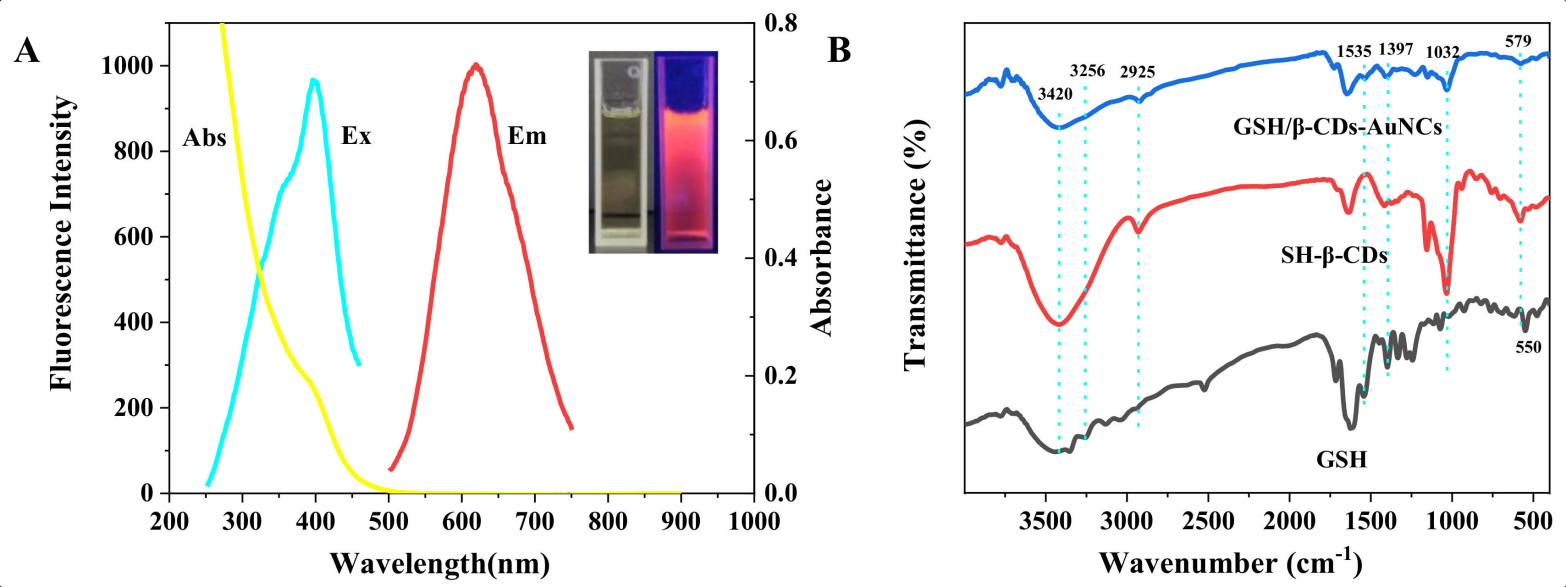
(**A**) Absorbance, excitation, and emission spectra of GSH/β-CDs-AuNCs. Insert image is GSH/β-CDs-AuNC solution under daylight (left) and UV light (right). (**B**) FTIR spectra of GSH, SH-β-CDs, and GSH/β-CDs-AuNCs, respectively.

As shown in Figure 1A, the synthesized GSH/β-CDs-AuNCs were pale yellow under daylight and emitted intense red fluorescence under UV light irradiation with the excitation of 365 nm. The GSH/β-CDs-AuNCs exhibited strong emission at 610 nm under excitation at 392 nm with a relatively large stoke shift of 218 nm. The absorption spectra of the GSH/β-CDs-AuNCs displayed only a weak shoulder peak at 400 nm, which was similar to reports from previous literature [19,43]. In the FTIR spectrum of GSH/β-CDs-AuNCs (Figure 1B), the peaks at 579 cm^−1^ and 550 cm^−1^ were assigned to the C–S stretching vibration. The characteristic peak at ~1032 cm^−1^ of the SH-β-CDs and GSH/β-CDs-AuNCs belongs to the coupled C–O/C–C stretching/O–H bending vibrations group of β-CDs [41]. The weak peak at ~3256 cm^−1^ observed in GSH and GSH/β-CDs-AuNCs was associated with N–H stretching, and the peak at ~1397 cm^−1^ was ascribed to C–N(O=C–NH) stretching. The above results from GSH/β-CDs-AuNCs showed the characteristic peaks of both GSH and SH-β-CDs, indicating the successful modification of both GSH and SH-β-CDs ligands on the surface of AuNCs.

**Figure 2:**
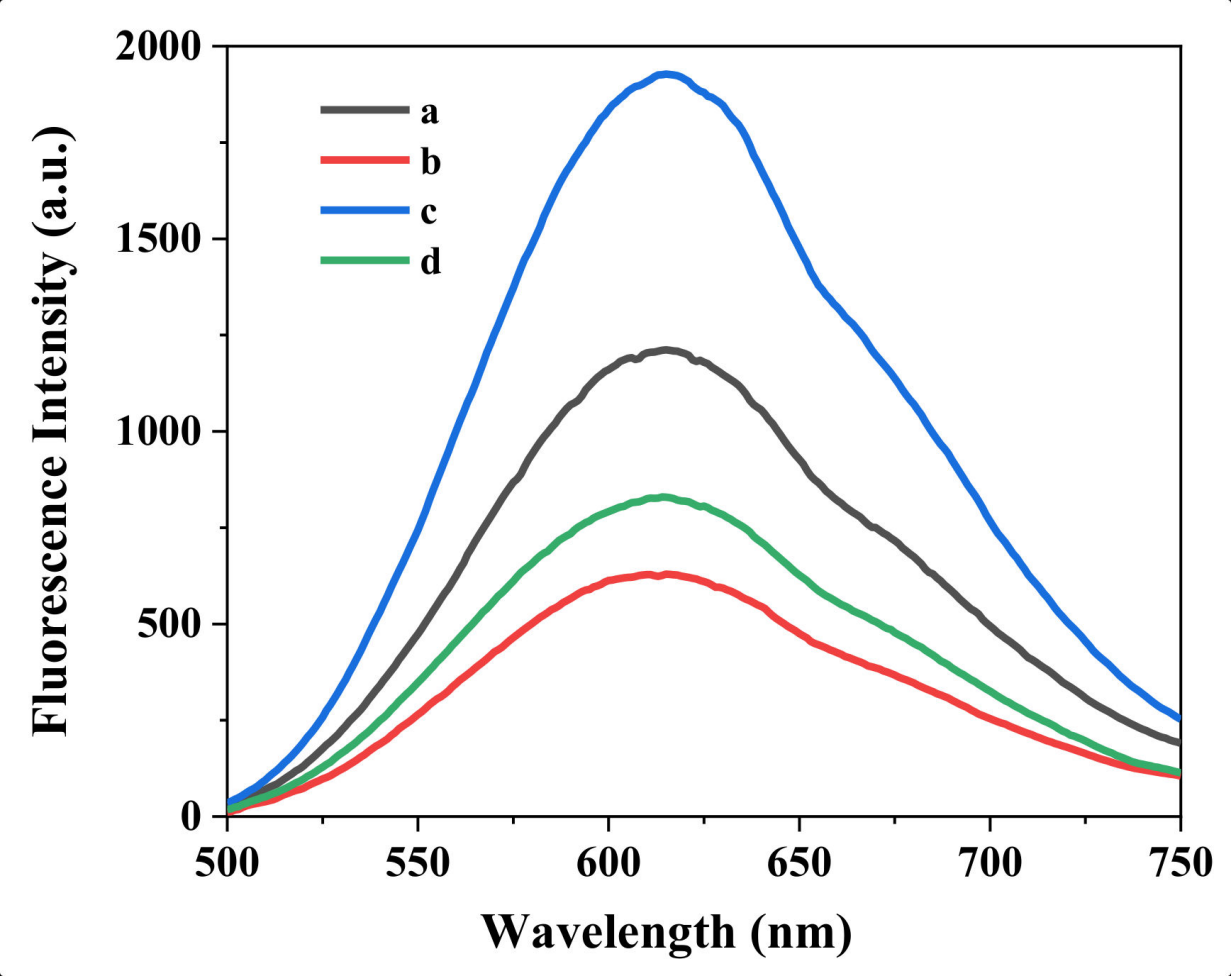
Fluorescence spectra of GSH-AuNCs and GSH/β-CDs-AuNCs in the absence and presence of paraquat. a, GSH-AuNCs; b, GSH-AuNCs in the presence of 500 ng/mL paraquat; c, GSH/β-CDs-AuNCs; d, GSH/β-CDs-AuNCs in the presence of paraquat.

**Figure 3:**
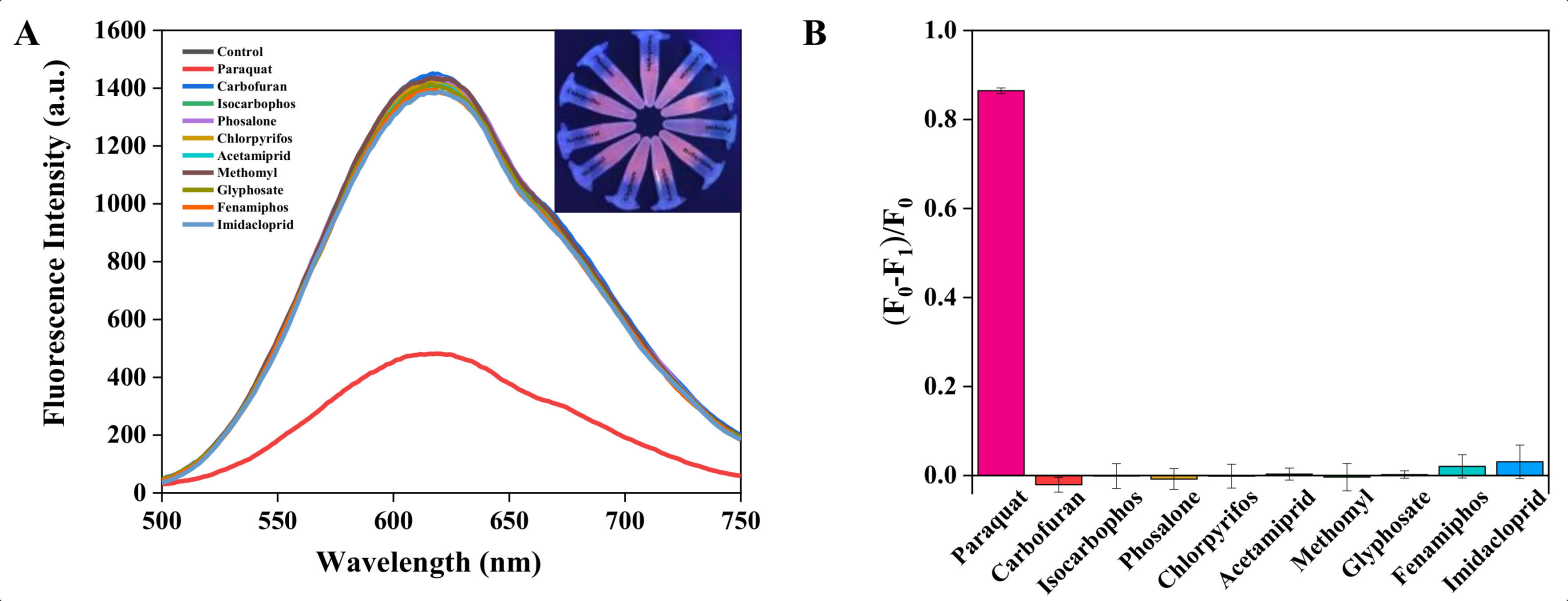
(**A**) Fluorescence spectra of GSH/β-CDs-AuNCs in the presence of various pesticides. Insert picture shows the corresponding inference assay excitation at 365 nm under UV light. (**B**) Quenching efficiency of AuNCs by various pesticides. All the pesticide concentrations were 500 ng/mL.

As shown in Figure 2, the fluorescence intensity of the GSH-AuNCs was enhanced 1.6-fold after the modification of β-CDs on the GSH-AuNCs surface. Moreover, the quenched fluorescence intensity (F_0_ − F, F_0_, and F are the fluorescence of AuNCs in the absence and presence of 500 ng/mL) was enhanced 1.4-fold after the modification of SH-β-CDs. Ten common pesticides (paraquat, carbofuran, isocarbophos, phosalone, chlorpyrifos, acetamiprid, methomyl, fenamiphos, and imidacloprid) were tested for interaction with GSH/β-CDs-AuNCs. As shown in Figure 3, paraquat quenched the fluorescence of the AuNCs by over 80%, compared to below 5% for the others, after incubation with the GSH/β-CDs-AuNCs. These results indicate that the GSH/β-CDs-AuNCs could be a good fluorescent probe for paraquat sensing.

### 3.2. Mechanism of Sensing

**Figure 4:**
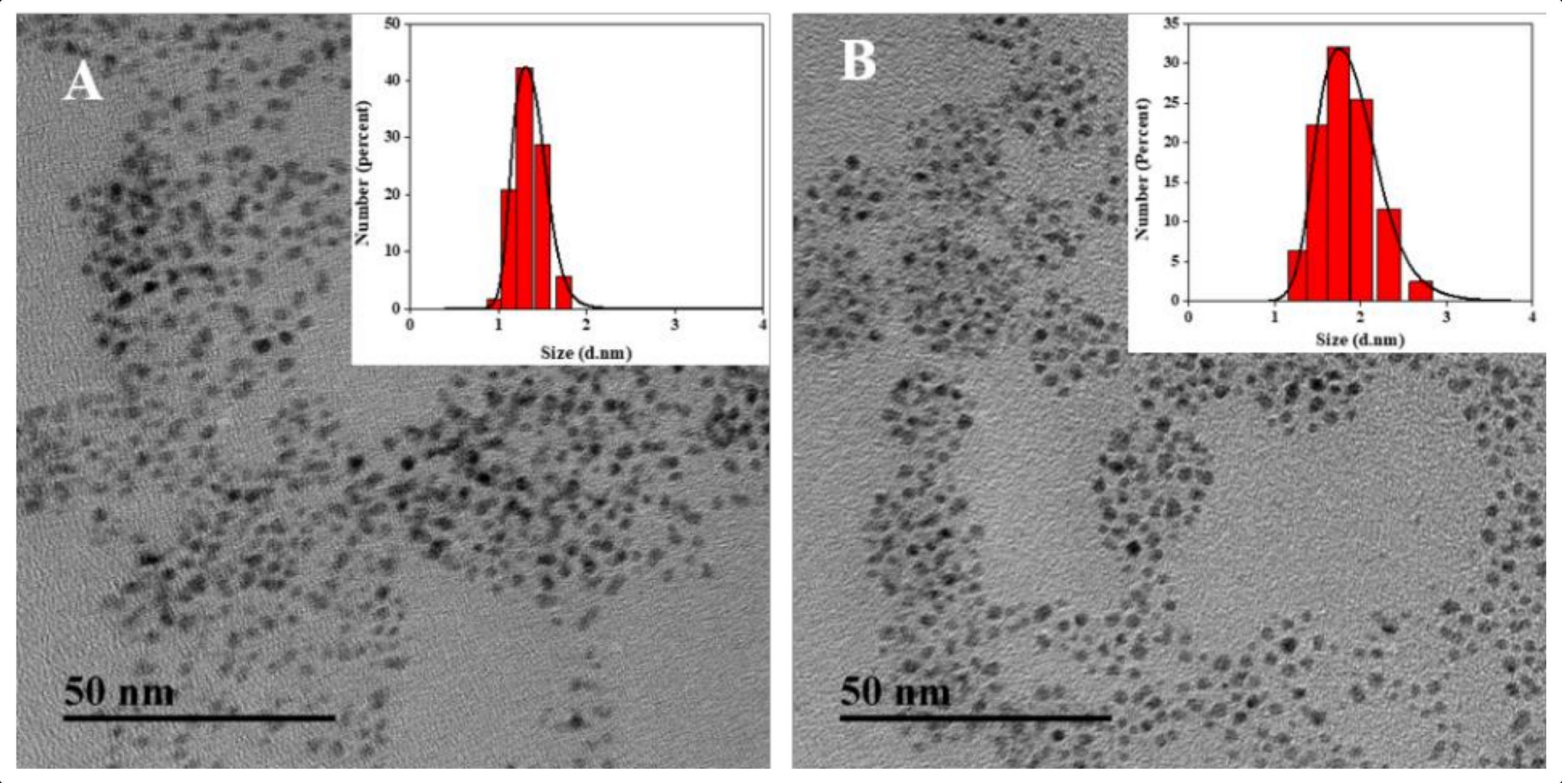
TEM images of GSH/β-CDs-AuNCs in the (**A**) absence and (**B**) presence of 500 ng/mL paraquat. Inset pictures show the corresponding hydrodynamic diameter of GSH/β-CDs-AuNCs.

To investigate the mechanism of fluorescence quenching, the GSH/β-CDs-AuNCs were analyzed by TEM and DLS. As shown in Figure 4A, GSH/β-CDs-AuNCs were spherical and dispersed with an average diameter of 1.37 ± 0.41 nm. The GSH/β-CDs-AuNCs remained monodispersed following the addition of paraquat. As shown in Figure 4B, the average diameter of AuNCs was 1.82 ± 1.3 nm in the presence of paraquat. The well-dispersed state of GSH/β-CDs-AuNCs indicated that the aggregation-induced quenching (AIQ) effect was not responsible for the GSH/β-CDs-AuNCs’ fluorescence quenching. The slightly increased hydrodynamic diameter of the GSH/β-CDs-AuNCs was probably caused by the binding with paraquat molecules. As shown in Figure S1, the zeta potential increased from −7.85 mV to −6.60 mV with addition of paraquat, indicating the existence of electrostatic binding between paraquat and the GSH/β-CDs-AuNCs.

Since the absorbance peak of paraquat (Figure S7) did not overlap with the excitation or emission spectra of GSH/β-CDs-AuNCs (Figure 1A), the inner filter effect (IFE) and resonance energy transfer (FRET) can be ruled out as causes of the fluorescence quenching by paraquat. Previous works have confirmed that the paraquat cation (PQ^2+^) can accept electrons from quantum dots (QDs) to form PQ^+^, accompanied by the appearance two absorption peaks ranged at 300–450 nm and 450–750 nm [44,45]. The surface ligand, GSH, on the GSH/β-CDs-AuNCs could be negatively charged owing to the abundant dissociated −COOH groups under alkaline conditions (pKa, 2.12, 3.59, 8.75, and 9.65). Thus, the most reasonable fluorescence-quenching mechanism is photoinduced energy transfer (PET), which has also been demonstrated in many GSH-capped quantum dot sensors [45,46,47,48]. Moreover, “host–guest” interactions between β-CDs and paraquat have been well studied [49], and could facilitate the quenching effect confined by spatial distance. This could also explain why the quenching efficiency was improved after modification with SH-β-CDs.

To better understand the quenching dynamics, the fluorescence lifetime was recorded as shown in Figure 4. The fluorescence lifetime decay was estimated at 610 nm after excitation at 392 nm. The decay curves were fitted properly with bi-exponential decay functions for GSH/β-CDs-AuNCs in the absence and presence of paraquat (Equation (1)) [50].

(1) $$I\left(t\right)=I_{0}+A_{1}e^{-\frac{t}{\Gamma_{1}}}+A_{2}e^{-\frac{t}{\Gamma_{2}}}$$

where I and I_0_ are the fluorescence intensities at times t and 0; A_1_ and A_2_ are exponential coefficients; and Γ_1_ together with Γ_2_ is the fluorescence decay time for the exponential components. The average lifetimes were calculated using Equation (2) [51].

(2) $$\Gamma =\frac{A_{1}\Gamma_{1}{}^{2}+A_{2}\Gamma_{2}{}^{2}}{A_{1}\Gamma_{1}+A_{2}\Gamma_{2}}$$

The GSH/β-CDs-AuNCs showed an average lifetime of 1.36 μs with components of 0.27 and 1.50 μs. In the presence of 500 ng/mL paraquat, the average lifetime of the GSH/β-CDs-AuNCs slightly decreased to 1.24 μs with components of 0.26 and 1.37 μs (Figure S2). Considering that the excited state lifetime in static quenching remained constant [50], the slightly changed lifetime in the presence of high concentrations of paraquat suggests that both of static and dynamic quenching effects could coexist.

### 3.3. Optimization of Paraquat Sensing

To realize the highest fluorescence enhancement, the final concentration of SH-β-CDs and temperature in β-CDs modification were investigated. As shown in Figure S4, the GSH/β-CDs-AuNCs demonstrated the highest emission with 100 µM SH-β-CDs at 50 °C. The incubation time was investigated as shown in Figure S5, and it was determined that the fluorescence quenching was completed after 5–10 min.

**Figure 5:**
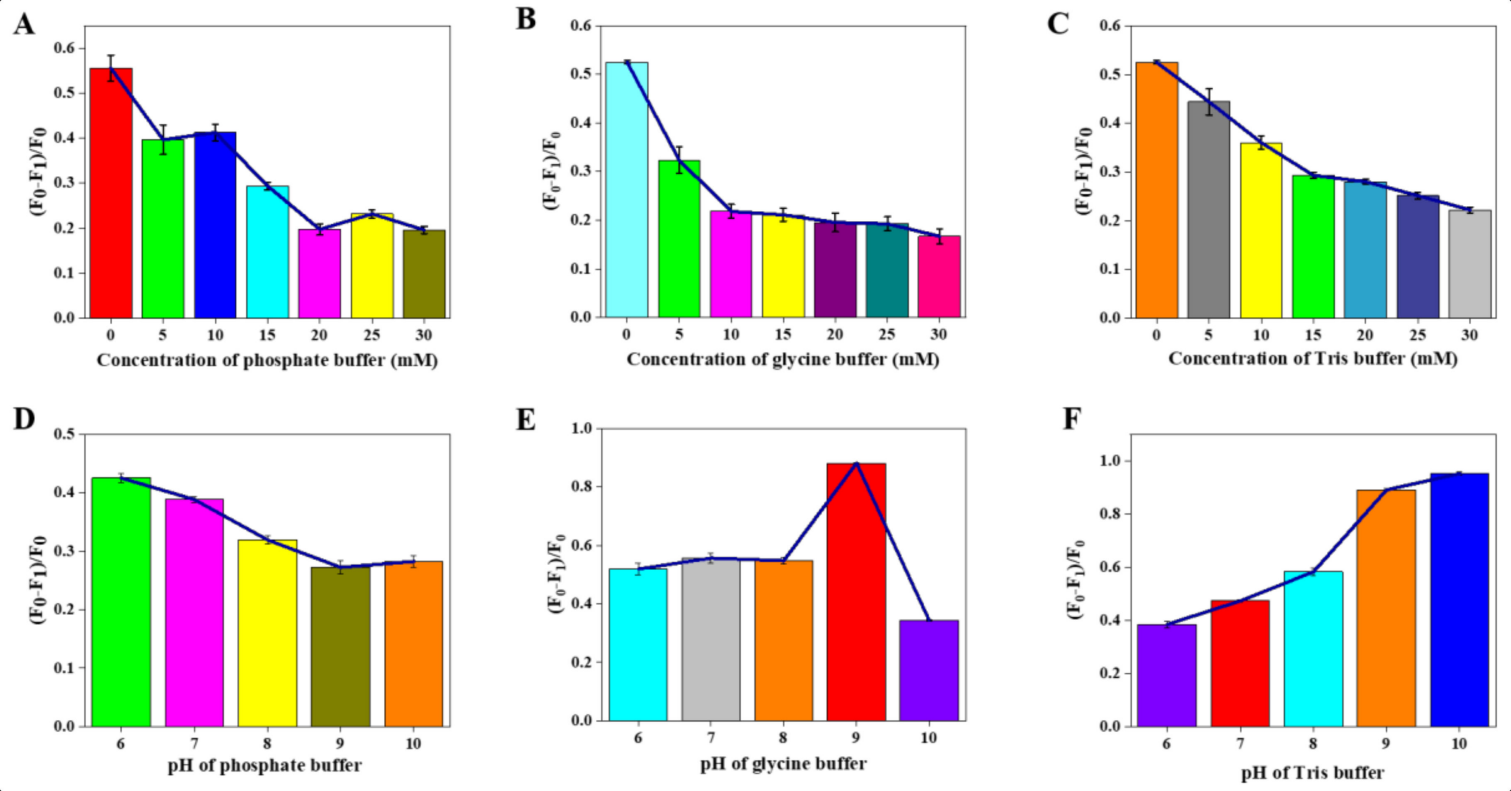
Effect of the concentration of (**A**) phosphate buffer, (**B**) glycine buffer, and (**C**) Tris-HCl buffer on quenching efficiency. Effect of the pH value of (**D**) phosphate buffer, (**E**) glycine buffer, and (**F**) Tris-HCl buffer on the quenching efficiency.

Three kinds of buffer were investigated in terms of the quenching efficiency of GSH/β-CDs-AuNCs. As shown in Figure 5A–C, the fluorescence-quenching efficiency [(F_0_ − F_1_)/F_0_] decreased with increasing concentration of the buffers. These results may have been due to the competition between cations and paraquat molecules to combine with GSH/β-CDs-AuNCs. Ultimately, 5 mM buffers were selected for further analysis due to good quenching efficiency.

The fluorescence-quenching efficiency changed significantly with the pH value variation. As shown in Figure 5D–F, the fluorescence-quenching efficiency showed different trends in the three kinds of buffers with pH value alteration. A possible reason is that different anions have different patterns of interaction with the paraquat molecule. In the above three buffers, only glycine can present in a zwitterion form when the pH value is less than pKa_2_ = 9.78 (-NH_2_) of glycine, and then act as a bridge to bind paraquat and GSH/β-CDs-AuNCs [52]. (The pKa values for the three buffers are listed in Figure S6.) As the pH value increased beyond 9.78, glycine molecules became more negatively charged, resulting in abruptly weakened quenching efficiency of paraquat. In Tris and Gly buffers, similar quenching ability of paraquat was demonstrated. Considering the buffer capacity of Tris was weak at pH 10, a Gly–NaOH buffer at pH 9.0 was eventually selected for further experiments.

### 3.4. Interference Analysis

**Figure 6:**
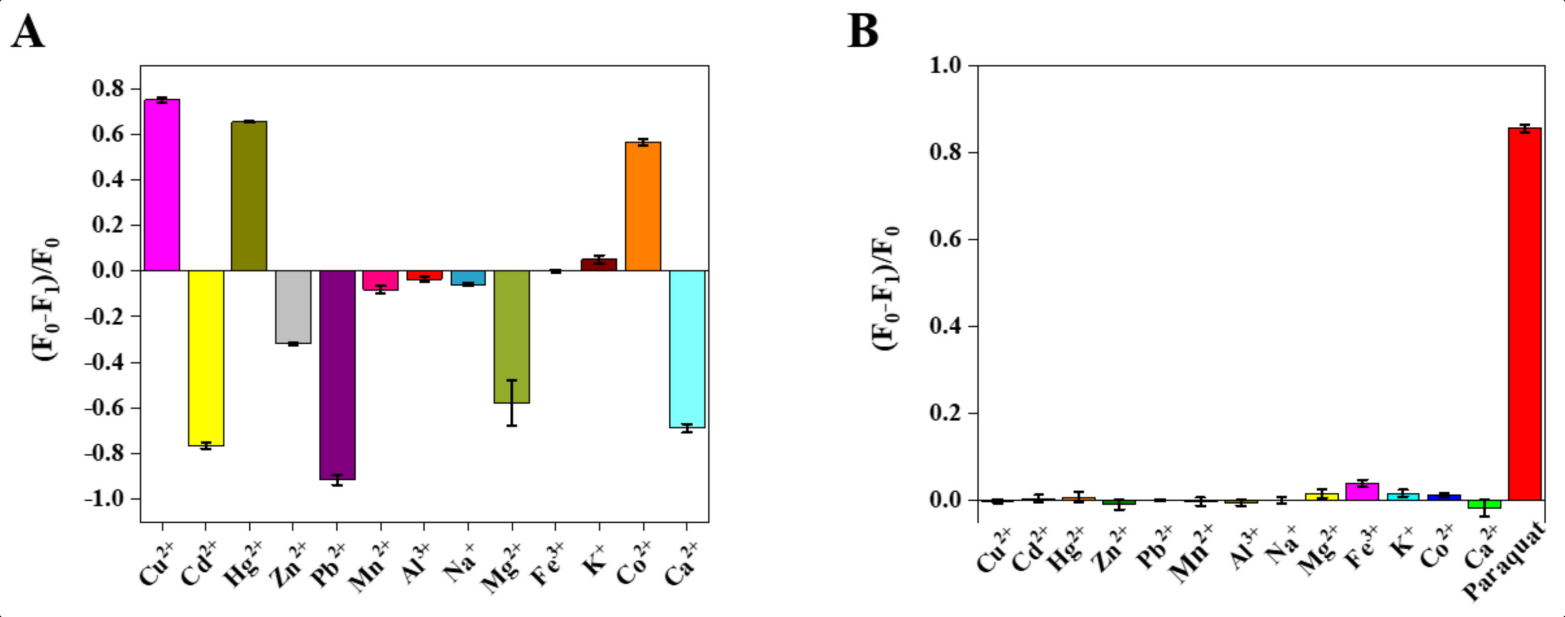
The interference evaluation of metal ions in paraquat sensing. (**A**) The response of metal ions to GSH/β-CDs-AuNCs. (**B**) The interference of metal ions in the presence of 100 μM EDTA. All the concentrations of metal ions, including Cu^2+^, Cd^2+^, Hg^2+^, Zn^2+^, Pb^2+^, Mn^2+^, Al^3+^, Na^+^, Mg^2+^, Fe^3+^, K^+^, Co^2+^, and Ca^2+^, were 10 μM.

The interference patterns of some common metal ions were evaluated since some works have reported that AuNCs respond to metal ions such as Zn^2+^ [53], Al^3+^ [54], Fe^3+^ [55], Cu^2+^, Hg^2+^, and Pb^2+^ [16]. The fluorescence intensity was recorded in the presence of various metal ions. The results showed that Cu^2+^, Hg^2+^, and Co^2+^ could quench the GSH/β-CDs-AuNCs while Cd^2+^, Pb^2+^, Mg^2+^, and Ca^2+^ enhanced the fluorescence intensity (Figure 6A). However, ethylene diamine tetraacetic acid (EDTA) could block these common metal ions and the subsequent interference from them was negligible (Figure 6B).

To better explain the good selectivity between paraquat and other involved pesticides shown in Figure 3, the UV–vis spectra of 10 pesticides and 9 pesticides’ chemical structures are listed in Figure S7A,B, respectively. The spectra of nine interference pesticides showed absorbance bands ranging from 200 to 350 nm, and the spectra did not overlap with the excitation of emission of the fluorescent GSH-AuNCs. Two main reasons could explain why the quenching behaviors of the other nine pesticides were quite different from that of paraquat: (1) lack of a binding group that can diminish the distance between pesticides and fluorescent AuNCs, which is essential for the FRET and PET effects [48]; (2) deficit in electron-accepting ability. Both these properties may be essential for paraquat’s quenching of the fluorescence of AuNCs. Hence, a good selective test between paraquat and other nine pesticides was obtained.

### 3.5. Analytical Performance Comparison between GSH-AuNCs, GSH/β-CDs-AuNCs, and Real Samples

**Figure 7:**
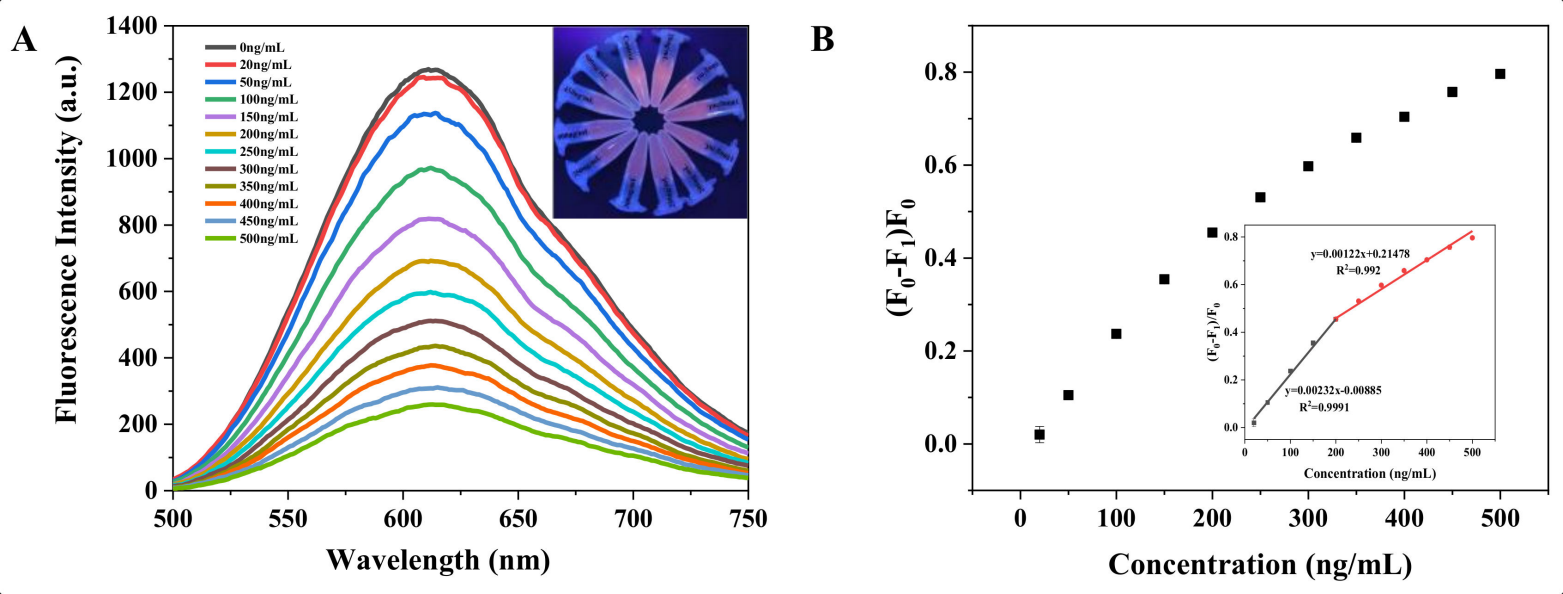
(**A**) Fluorescence spectra under various concentrations of paraquat. The inset picture is the fluorescence photo showing the detection of various concentrations of paraquat with GSH-AuNCs. (**B**) Calibration curve of paraquat sensing with different concentrations of 0, 5, 10, 20, 50, 100, 150, 200, 250, 300, 350, 400, 450, and 500 ng/mL, respectively. The inset shows the two linear fitting curves between the fluorescence-quenching efficiency [(F_0_ − F_1_)/F_0_] and the paraquat concentrations.

**Figure 8:**
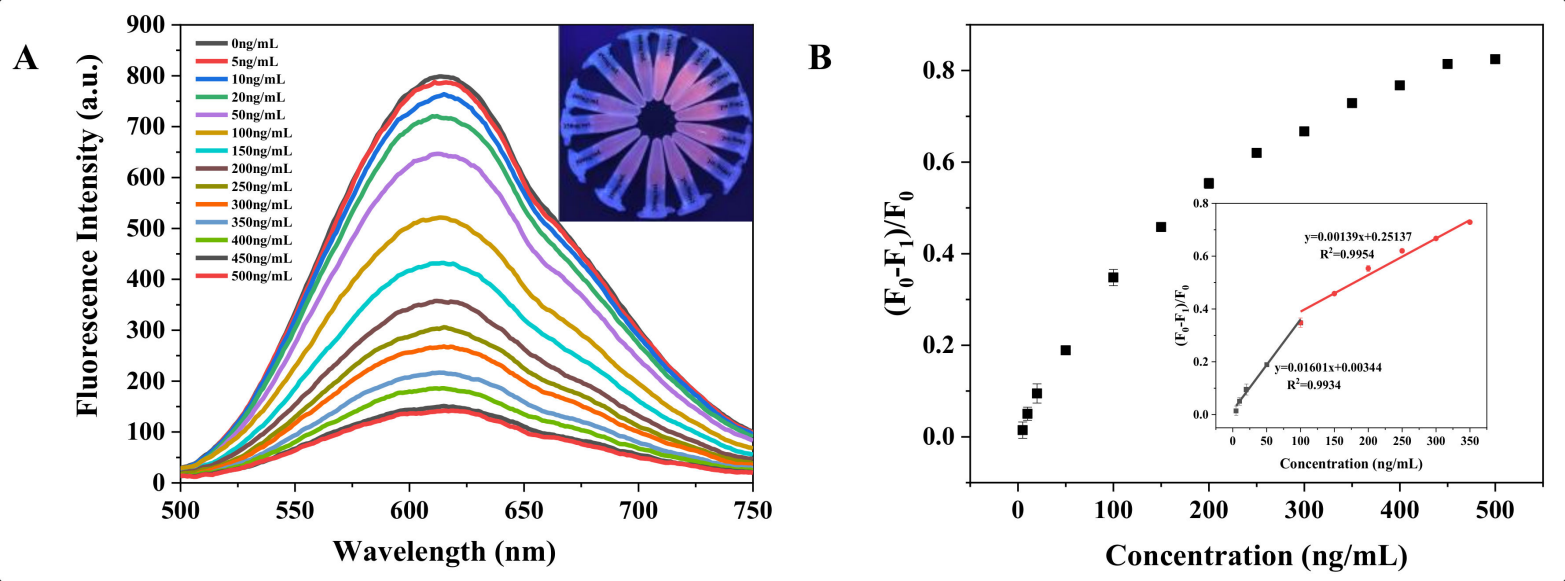
(**A**) Fluorescence spectra under various concentration of paraquat. The inset picture is the fluorescence photo showing the detection of various concentrations of paraquat with GSH/β-CDs-AuNCs. (**B**) Calibration curve of paraquat sensing with different concentrations of 0, 5, 10, 20, 50, 100, 150, 200, 250, 300, 350, 400, 450, and 500 ng/mL, respectively. The inset shows the two linear fitting curves between the fluorescence-quenching efficiency [(F_0_ − F_1_)/F_0_] and the paraquat concentrations.

The quantitative performance of the proposed fluorescence assay between GSH-AuNCs and GSH/β-CDs-AuNCs was evaluated. The fluorescence spectra of GSH-AuNCs and GSH/β-CDs-AuNCs with various concentrations of paraquat are shown in Figure 7A and Figure 8A, respectively. The fluorescence intensity of the AuNCs decreased dramatically with the increasing paraquat concentration. Two good linear curves between the fluorescence quenching efficiency [(F_0_ − F_1_)/F_0_] and the paraquat concentration were obtained with GSH-AuNCs and GSH/β-CDs-AuNCs. With GSH-AuNCs, the linear ranges were 20–200 ng/mL (R^2^ = 0.9991) and 200–500 ng/mL (R^2^ = 0.9921), respectively (Figure 7B). The limit of detection (LOD) was calculated to be 6.2 ng/mL according to 3S/N [56]. In the case of GSH/β-CDs-AuNCs, the linear ranges were 5–100 ng/mL (R^2^ = 0.9934) and 100–350 ng/mL (R^2^ = 0.9954) (Figure 8B). The limit of detection (LOD) was calculated to be 1.2 ng/mL.

The above results demonstrate that the sensitivity of paraquat sensing was obviously improved by β-CDs modification of GSH-AuNCs. The increase in sensitivity was ascribed to the fluorescence enhancement of GSH-AuNCs and the “host–guest” interaction between paraquat and the β-CDs on the GSH/β-CDs-AuNC surfaces. Compared with recently reported typical methods for paraquat detection, our proposed method is more sensitive (Table 1).

To confirm the reliability of the proposed method, tap water from the local water supply system and lake water from Taihu Lake in Wuxi were collected and tested using our method. The lake water samples were spiked with 50, 150, and 300 ng/mL paraquat. Additionally, Chinese cabbage samples were spiked with 100, 150, and 300 ng/mL paraquat, values which are all lower than the American residue restriction for paraquat in vegetables (0.5 ppm), and then diluted 5-fold. The above samples were then tested using the GSH/β-CDs-AuNC-based method. Recoveries between 93.6% and 103.8% were obtained (Table S1), which confirmed the reliability of this method for paraquat detection in practical applications.

## 4. Conclusions

A novel fluorescent method for paraquat detection based on GSH/β-CDs-AuNCs was developed. The established method demonstrated low cost and high sensitivity and selectivity. The mechanism of paraquat sensing was via the fluorescence quenching by photoinduced energy transfer (PET) effect between the GSH/β-CDs-AuNCs and paraquat. The GSH/β-CDs-AuNC-based sensor demonstrated good accuracy in paraquat detection in real samples. Regarding host molecules on the surface of nanomaterials able to facilitate the binding between target molecules and nanosensors, host molecule modification of fluorescent metal nanoclusters might enhance their relevance for further investigation in various sensors.

## Figures and Tables

**Table 1:** Comparison between our method and the reported fluorescence analysis methods of paraquat.

Analytical Materials	Limit of Detection (LOD)	Linear Range	Ref.
Sodium montmorillonite clay	0.37 µM	2.0–8.0 µM	[57]
Fluorescent dye	0.270 µM	1.00–100 µM	[58]
PP6@graphene	0.06 µM	0.2–2 μM and 2–18μM	[59]
N-GQDs	73.9 nM	0.05–2.0 μg/mL	[60]
OVA-AuNCs	49 nM	0.2 to 1000 µM	[61]
PWP5-rGO	3.5 nM	0.01–2.0 and 2.0–50.0 µM	[62]
CdS QDs	38.8 nM	25–1500 ng/mL	[47]
Squaraines	372 nM	0–140 μM	[63]
Pyranine	0.2 μM	1.0–20.0 μM	[64]
GSH/β-CDs-AuNCs	1.2 ng/mL (4.67nM)	5.0–350 ng/mL	Our work

## Supplementary Materials

The following are available online at https://www.mdpi.com/article/10.3390/foods10061178/s1, Figure S1. The zeta potential of AuNCs 1, which are GSH/β-CDs-AuNCs dispersed in ultrapure water; AuNCs 2, which are GSH/β-CDs-AuNCs diluted with Gly–NaOH buffer; and AuNCs 3, which are GSH/β-CDs-AuNCs mixed with Gly–NaOH and 300 ng/mL paraquat. Figure S2. Time-resolved fluorescence spectra of GSH/β-CDs-AuNCs in the (A) absence and (B) presence of 500 ng/mL paraquat. Figure S3. Stern–Volmer plot for the fluorescence of GSH/β-CDs-AuNCs quenched by different concentrations of paraquat. F_0_ and F_1_ are the fluorescence intensity of GSH/β-CDs-AuNCs in the absence and presence of paraquat, respectively. Figure S4. The modification of GSH-AuNCs with (A) different concentrations of SH-β-CDs and (B) under different temperatures. Figure S5. Paraquat quenching efficiency on GSH/β-CDs-AuNCs fluorescence with different incubation times, under incubation condition: 400 rpm, 25 °C. Figure S6. The chemical structure and acidity coefficients of three buffers. Figure S7. (A) The absorbance spectra and (B) chemical structures of 10 different pesticides including paraquat, carbofuran, isocarbophos, phosolone, chlorpyrifos, acetamiprid, methonmyl, glyphosate, fenamiphos, and imidacloprid. Table S1: Different spiked samples recovery detection assay.
