# Supplementary material for: A Fluorescent Detection for Paraquat Based on β-CDs-Enhanced Fluorescent Gold Nanoclusters
Source: Foods. 2021 May 24;10(6):1178. doi: 10.3390/foods10061178 (PMC8225061; doi:10.3390/foods10061178)
Supplement: Supplementary file 1 [file foods-10-01178-s001.zip › foods-1210016-supplementary.pdf]

Supporting information

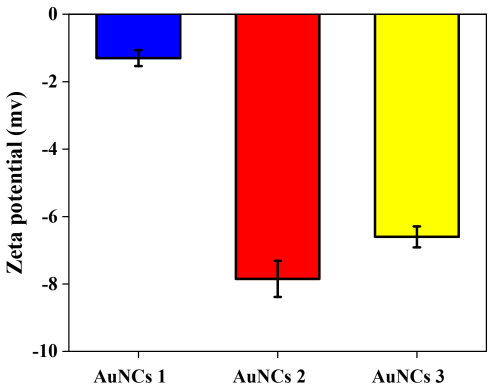

Figure S1. The zeta potential of AuNCs 1, which is GSH/β-CDs-AuNCs dispersed in ultrapure water. AuNCs 2, which is GSH/β-CDs-AuNCs diluted with Gly-NaOH buffer, and AuNCs 3, which is GSH/β-CDs-AuNCs mixed with Gly-NaOH and 300ng/mL paraquat.

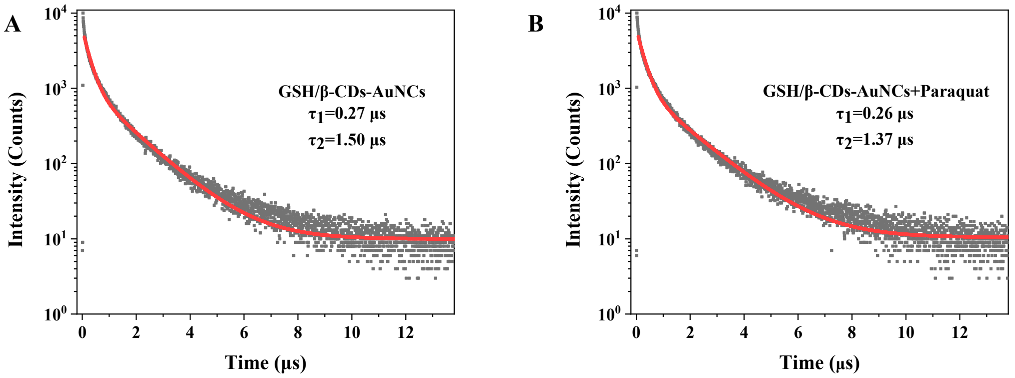

Figure S2. Time-resolved fluorescence spectra of GSH/β-CDs-AuNCs in (A) absence and (B) presence of 500ng/mL paraquat.

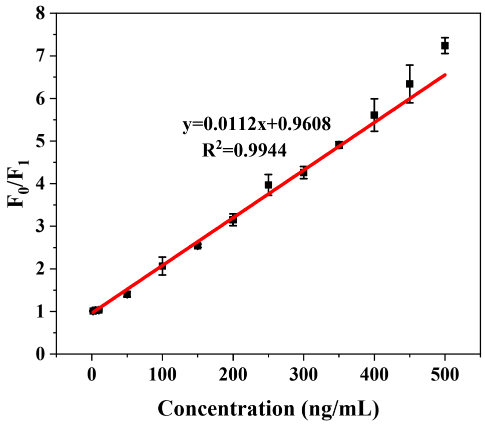

Figure S3. Stern−Volmer plot for the fluorescence of GSH/β-CDs-AuNCs quenched by different concentrations of paraquat. F0 and F1 were fluorescence intensity of GSH/β-CDs-AuNCs in the absence and presence of paraquat, respectively.

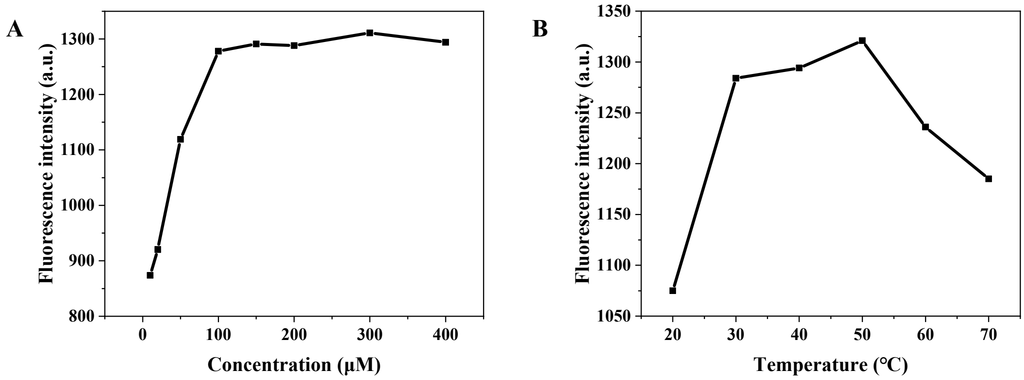

Figure S4. The modification GSH-AuNCs with (A) different concentration of SH-β-CDs and (B) under different temperature.

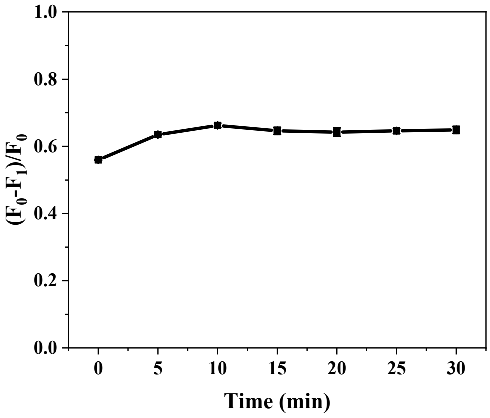

Figure S5. The paraquat quenching efficiency on GSH/β-CDs-AuNCs fluorescence with different incubation time, under incubation condition: 400rpm, 25℃.

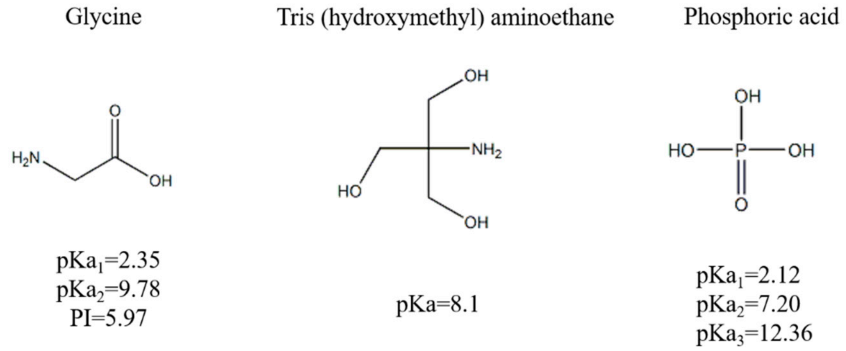

Figure S6. The chemical structure and acidity coefficient of three buffer.

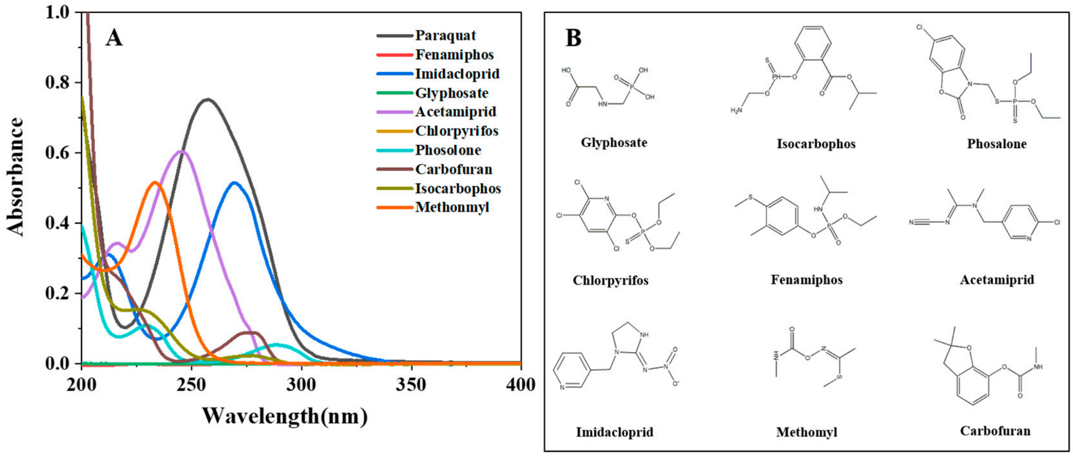

Figure S7. (A)The absorbance spectrum and (B) Chemical structure of 10 kinds pesticides which include paraquat, carbofuran, isocarbophos, phosolone, chlorpyrifos, acetamiprid, methonmyl, glyphosate, fenamiphos, and imidacloprid.

Table S1. Different spiked samples recovery detection assay.

Sample (Type)	Spiked concentration (ng/mL）	Concentration found（ng/mL)	Recovery (%)	Standard deviation (%)
Lake water	50	50.75	101.5	3.92
	150	149.0	99.3	2.53
	300	292.5	97.5	2.55
Chinese cabbage	100	93.6	93.6	5.22
	150	156.2	103.8	4.82
	300	293.4	97.8	2.83
